# Supplementary material for: Differential Small RNA Responses against Co-Infecting Insect-Specific Viruses in Aedes albopictus Mosquitoes
Source: Viruses. 2020 Apr 21;12(4):468. doi: 10.3390/v12040468 (PMC7232154; doi:10.3390/v12040468)
Supplement: Supplementary file 1 [file viruses-12-00468-s001.pdf]

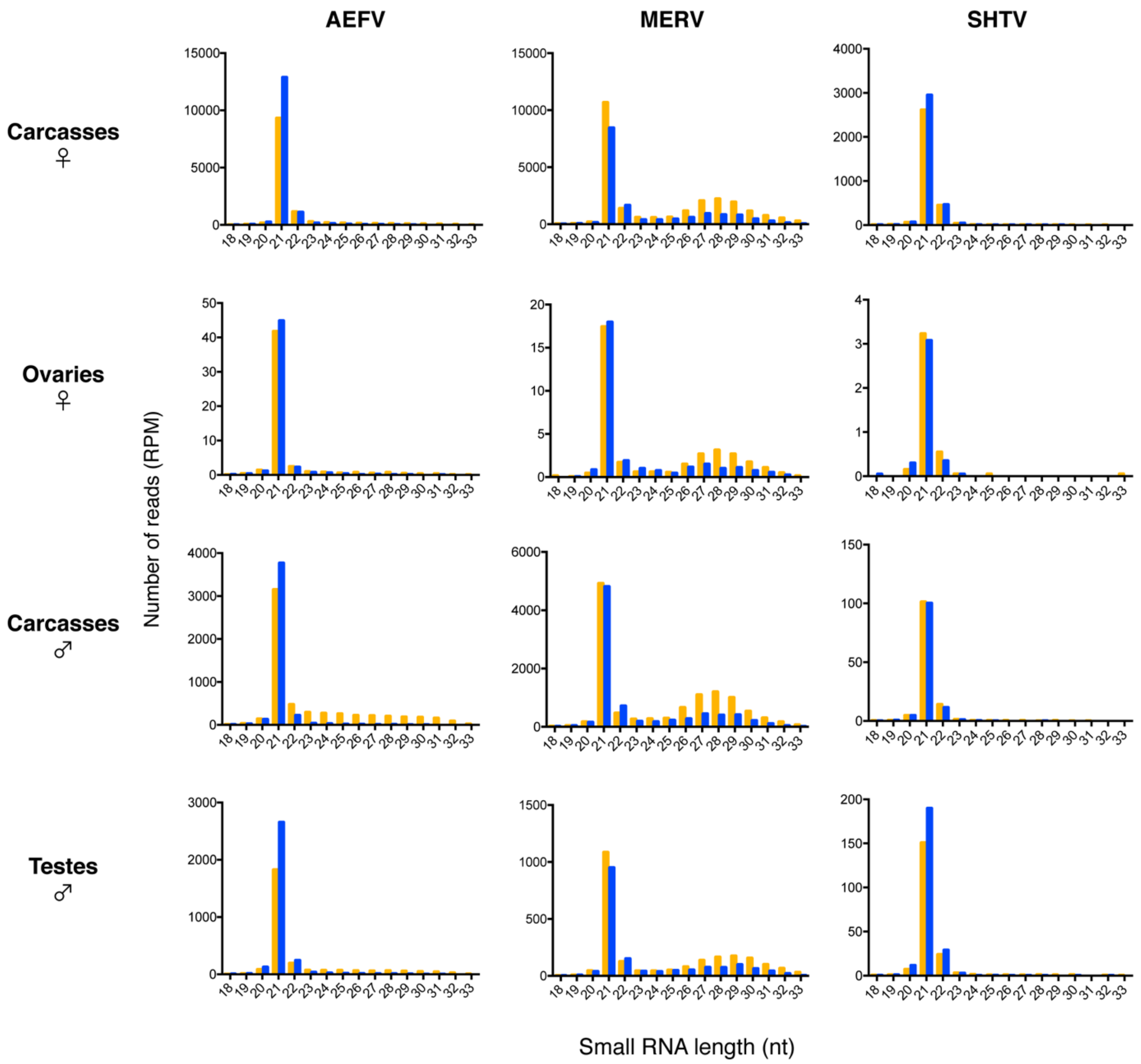

Figure S1. Size distribution of small RNAs mapped to ISVs in reproductive tissues and carcasses of female or male *Ae. albopictus* mosquitoes. Yellow and blue bars represent positive- and negative-stranded reads, respectively.

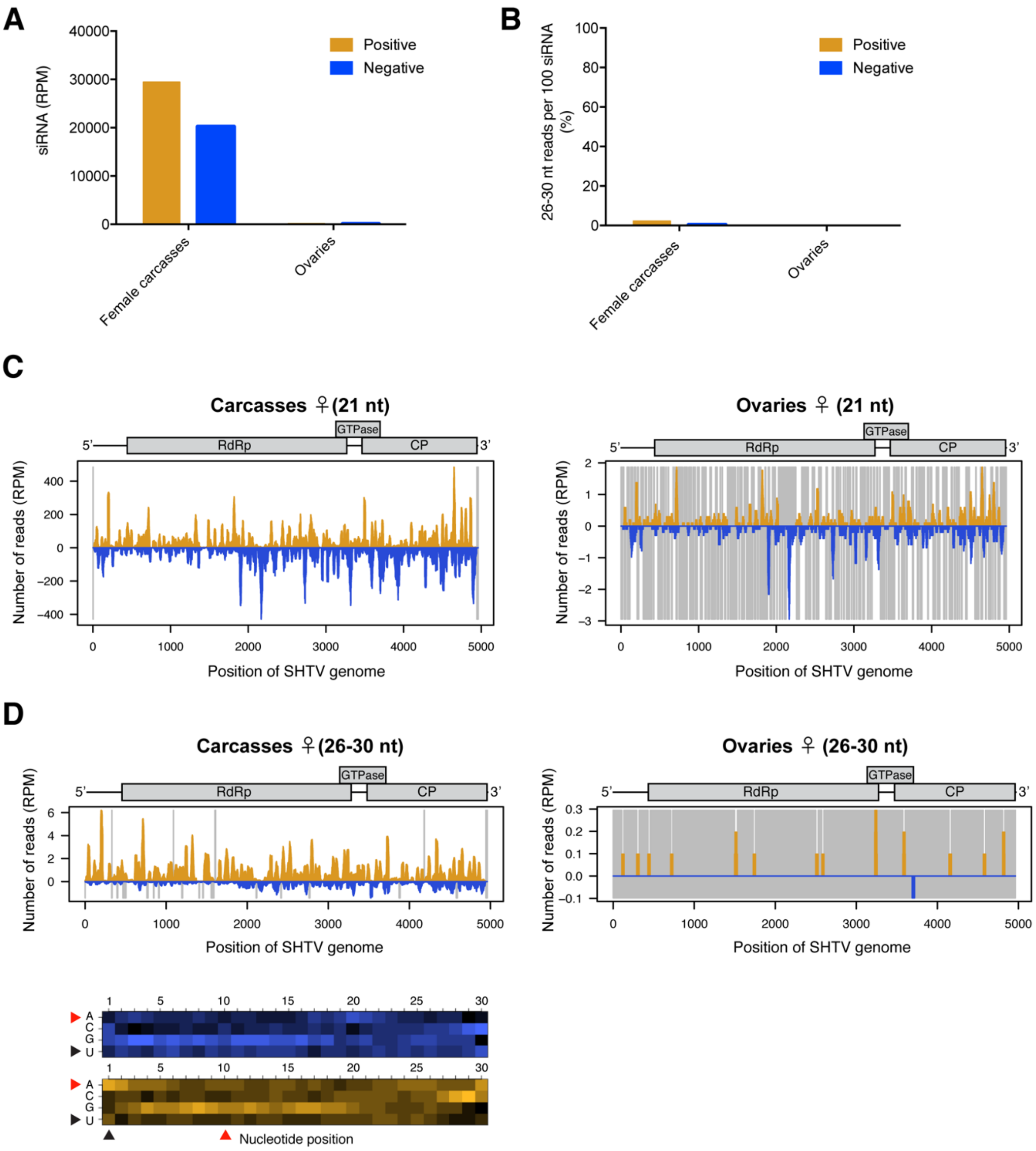

Figure S2. Small RNA profile of SHTV in reproductive tissues and carcasses of female *Ae. albopictus*-Japan mosquitoes. Normalized siRNA reads per one million (RPM) mapped to the SHTV genome is shown in (A). Proportion of 26-30 nt long SHTV-derived small RNAs per 100 vsiRNAs is shown in (B). The distribution of siRNA (21 nt) (C) and piRNA-like small RNAs (26-30 nt) (upper panel, D) mapped to the SHTV genome is shown. Yellow and blue bars represent positive- and negative-stranded reads, respectively. Regions with no coverage are indicated by gray lines. For 26-30 nt SHTV-derived piRNA-like small RNAs detected in carcasses, relative nucleotide frequency at each position is shown as a heat map (lower panel in D), in which the color intensity denotes the frequency. 26-30 nt small RNA reads were not detected in ovary samples (B). The black and red arrowheads point to the 1U and 10A positions, respectively.

Table S1. Primer sequences used.

Purpose	Sequences
AEFV_NS4 RNA standard	Forward: GGATCCTAATACGACTCACTATAGGAGTTCAGCTAGTCTGTACGACATCC
	Reverse: GCGAAATTTACTGAAAGGGGCCATAG
qPCR for AEFV	Forward: AGGCACAGTTGGAGGGTTCC
	Reverse: TACAACGCTGGGAAGGCCAA
qPCR for MERV	Forward: GAGGTGTCGGATAAATTTCTAG
	Reverse: TTATCTGATAGGTGCCCCTTC
qPCR for SHTV	Forward: TTCTTAGGAATGAGGCTCATG
	Reverse: ACTAGGTCGTTGGGGCTCTC
qPCR for DCR2	Forward: CCAAAACCGCTGAAGGAGA
	Reverse: CTGGACAATCAATCCGAGCA
qPCR for Ago2	Forward: GTCGGGTGGATTTGGTGTTC
	Reverse: TATCGCCCCGTTTCCTATCC
qPCR for Piwi1-4	Forward: CGACACGAACGACAAATCCA
	Reverse: GGTACTCGTTGAGCGCCTTG
qPCR for Piwi5/6	Forward: CGCAGTTGGTGATGTGTGTG
	Reverse: ATGACTTGCGTGGGAATGG
qPCR for Piwi7	Forward: CTGAAGACCCGAACGATCAC
	Reverse: TTACCATCACCGAAGCCAGA
qPCR for Piwi8/9	Forward: GCAGTGGTTCGCAGTGGTTC
	Reverse: CCGGCGAATCGTTGGGAATG
qPCR for Ago3	Forward: CCATTCGCCGGACATTCTGC
	Reverse: TACTGACAGCAAGCGGGGAC

Table S2. P values for the qPCR analysis depicted in Figure 6 normalized with actin

	**Carcass_♀ vs Ovaries**	**Carcass_♂ vs Testes**	**Carcass_♀ vs Carcass_♂**	**Ovaries vs Testes**	**Ovaries vs Carcass_♂**	**Carcass_♀ vs Testes**
DCR2	1,49E-04	1,98E-03	1,85E-02	1,23E-04	< 0,0001	6,05E-01
Ago2	5,90E-04	3,61E-01	1,94E-02	1,41E-04	1,33E-04	2,66E-02
Piwi1-4	9,71E-04	3,16E-03	9,15E-01	1,22E-03	9,71E-04	3,16E-03
Piwi5/6	< 0,0001	8,86E-01	2,46E-02	< 0,0001	< 0,0001	2,43E-02
Piwi7	< 0,0001	5,20E-03	2,81E-01	< 0,0001	< 0,0001	1,89E-01
Piwi8/9	6,06E-03	2,91E-02	9,41E-01	3,15E-03	5,96E-03	2,61E-02
Ago3	4,96E-04	1,30E-03	1,22E-02	5,14E-04	4,87E-04	1,29E-02

Table S3. P values for the qPCR analysis depicted in Figure 6 normalized with RPL18

	**Carcass_♀ vs Ovaries**	**Carcass_♂ vs Testes**	**Carcass_♀ vs Carcass_♂**	**Ovaries vs Testes**	**Ovaries vs Carcass_♂**	**Carcass_♀ vs Testes**
DCR2	1,32E-03	8,23E-01	3,37E-03	6,32E-04	6,19E-04	4,09E-03
Ago2	5,74E-03	1,88E-02	1,94E-02	< 0,0001	< 0,0001	9,55E-03
Piwi1-4	5,75E-04	3,52E-03	9,99E-01	6,93E-04	5,75E-04	3,52E-03
Piwi5/6	< 0,0001	1,58E-02	9,03E-03	< 0,0001	< 0,0001	2,89E-03
Piwi7	< 0,0001	2,46E-01	1,38E-01	< 0,0001	< 0,0001	2,50E-01
Piwi8/9	8,92E-03	2,76E-03	6,58E-01	5,31E-04	3,13E-03	1,85E-02
Ago3	3,06E-04	1,15E-02	2,63E-02	3,03E-04	2,93E-04	5,71E-01
